# Economic threat heightens conflict detection: sLORETA evidence

**DOI:** 10.1093/scan/nsaa139

**Published:** 2020-10-07

**Authors:** Kyle Nash, Alex Tran, Josh Leota, Andy Scott

**Affiliations:** Department of Psychology, University of Alberta, Edmonton, Canada; Department of Psychology, University of Alberta, Edmonton, Canada; Department of Psychology, University of Alberta, Edmonton, Canada; Department of Psychology, University of Alberta, Edmonton, Canada

**Keywords:** economic threat, conflict detection, N2, anterior cingulate cortex

## Abstract

Economic threat has far-reaching emotional and social consequences, yet the impact of economic threat on neurocognitive processes has received little empirical scrutiny. Here, we examined the causal relationship between economic threat and conflict detection, a critical process in cognitive control associated with the anterior cingulate cortex (ACC). Participants (*N* = 103) were first randomly assigned to read about a gloomy economic forecast (Economic Threat condition) or a stable economic forecast (No-Threat Control condition). Notably, these forecasts were based on real, publicly available economic predictions. Participants then completed a passive auditory oddball task composed of frequent standard tones and infrequent, aversive white-noise bursts, a task that elicits the N2, an event-related potential component linked to conflict detection. Results revealed that participants in the Economic Threat condition evidenced increased activation source localized to the ACC during the N2 to white-noise stimuli. Further, ACC activation to conflict mediated an effect of Economic Threat on increased justification for personal wealth. Economic threat thus has implications for basic neurocognitive function. Discussion centers on how effects on conflict detection could shed light on the broader emotional and social consequences of economic threat.

Economic threat has a variety of emotional and social consequences. For example, poor economic prospects have been associated with higher levels of stress, anxiety, depression and suicide (Brown *et al.*, 2005; Jenkins *et al.*, 2008; Stevenson and Wolfers, 2008; Diener *et al.*, 2010; Fitch *et al.*, 2011; Bechtel, 2012). Economic threat has also been associated with increases in authoritarianism, prejudice, anti-immigrant sentiment and nationalism (Adorno *et al.*, 1950; Baughn and Yaprak, 1996; Rickert, 1998; Durvasula and Lysonski, 2006; Stevens *et al.*, 2006; Butz and Yogeeswaran, 2011; Goldstein and Peters, 2014; Burhan and van Leeuwen, 2016; Rothwell and Diego-Rosell, 2016; Gidron and Hall, 2017; Miller, 2017; Fabian *et al.*, 2020). Despite these varied and far-reaching psychological effects, the impact of economic threat on basic neurocognitive mechanisms remains unclear. The examination of such fundamental processes may be particularly salient because the 2007–09 Great Recession, which began with the subprime mortgage crisis in the United States and spread to much of Europe and elsewhere, had a profound impact on emotional and social outcomes (Roszkowski and Davey, 2010; Karanikolos *et al.*, 2013; McInerney *et al.*, 2013; Buffel *et al.*, 2015; Kriesi and Pappas, 2015; Aslanidis, 2016; Bianchi, 2016; Margerison-Zilko *et al.*, 2016; Ybarra *et al.*, 2016). As of writing, increasing economic angst looms again amidst the COVID-19 pandemic. Understanding the effect of economic threat on neurocognitive processes could help shed light on the mechanisms involved in these broader psychological consequences.

Here, we examined if economic threat has an impact on conflict detection. Conflict detection, or the recognition of coactivated, competing cognitive representations, constitutes the first step in cognitive control processes (Carter and Van Veen, 2007; Inzlicht *et al.*, 2015). Conflict detection is primarily associated with the dorsal anterior cingulate cortex (ACC), which is thought to recruit the lateral prefrontal cortex (PFC) for top-down control and conflict resolution (Botvinick *et al.*, 2004; MacDonald *et al.*, 2000; Mansouri *et al.*, 2009; Miller and Cohen, 2001; Yeung *et al.*, 2004). For example, functional magnetic resonance imaging (fMRI) studies reveal that dorsal ACC activation to conflict predicts lateral PFC activation and behavioral adaptation (Badre and Wagner; 2004; Botvinick *et al.*, 2004; Kerns *et al.*, 2004). Similarly, electrophysiological studies reveal that a family of event-related potential (ERP) components elicited by different conflicts—namely, the N2 (or N200), the error-related negativity (ERN or Ne) and the feedback-related negativity (FRN)—have all been source localized to the dorsal ACC (Cavanagh and Shackman, 2015), and mean amplitude in these conflict-related components often predicts behavioral adjustments or better performance, i.e. conflict resolution (Gehring *et al.*, 1993; Luu *et al.*, 2003; Nieuwenhuis *et al.*, 2003).

Though foundational models of ACC functional organization subdivided the ACC into emotional-rostral and cognitive-dorsal regions (e.g. Bush *et al.*, 2000), contemporary models recognize the dorsal ACC as a cortical hub involved in integrating cognitive and emotional processes (Critchley *et al.*, 2013; Cromheeke and Mueller, 2014; Egner *et al.*, 2008; Shackman *et al.*, 2011; Inzlicht *et al.*, 2015). For example, the adaptive control model posits that negative affect, pain and cognitive control engage a domain-general process to solve comparable problems—i.e. these domains act as signals of conflict or uncertainty in current goal-pursuits that may require adaptive changes in attention and behavior (Shackman *et al.*, 2011). A common neurobiological process provides a mechanistic explanation of why negative affect, pain and cognitive control can all modulate each other.

Economic threat, as an anxiety-provoking and uncertain experience, should cause dorsal ACC activation and increase conflict detection sensitivity. This reasoning is consistent with neuropsychological theory and research in which anxiety and uncertainty function to increase sensitivity to aversive stimuli and increase avoidance motivation (Gray and McNaughton, 2000; McNaughton and Corr, 2004; Hirsh *et al.*, 2012). Indeed, anxiety and uncertainty are associated with larger amplitudes in electrophysiological markers of conflict. For example, higher trait sensitivity to punishment and negative arousal is associated with larger FRN amplitudes to monetary loss (De Pascalis *et al.*, 2010). Negative emotionality is also associated with larger FRN amplitudes and increased source-localized ACC activation during the FRN timeframe (Santesso *et al.*, 2012). Higher trait anxiety is associated with larger ERN amplitudes (Aarts and Pourtois, 2010; Moser *et al.*, 2013). Higher levels of both state and trait anxiety are associated with larger N2 amplitudes (Righi *et al.*, 2009; Sehlmeyer *et al.*, 2010).

To our knowledge, no research has experimentally examined the causal impact of economic threat on ACC functioning in conflict monitoring. Here, we first randomly assigned participants to either an economic threat or a control condition. After the manipulation, we used electroencephalogram (EEG) to index conflict detection processes to infrequent blasts of white noise (i.e. startle stimuli) in a passive auditory oddball task. This task elicits a characteristic ERP, namely an N2–P3 complex, in which the N2 component reflects conflict detection between frequent, expected and neutral auditory stimuli and infrequent, unexpected and aversive auditory stimuli (Patel and Azzam, 2005). To index ACC activation during conflict detection processes, we used standardized low-resolution brain electromagnetic tomography (sLORETA; Pascual-Marqui, 2002) on 64-channel EEG to source localize the effect of economic threat versus a control condition on whole brain activation during the N2 component. We hypothesized that economic threat would cause increased activation in the dorsal ACC during the N2 timeframe, indicating heightened sensitivity to conflict.

Notably, conflict sensitivity is frequently cited as a key mechanism in political, moral and group-based ideologies. For example, according to the model of motivated social cognition, increased sensitivity to uncertainty and threat compels people to seek psychological sanctuary in ‘black-and-white’ beliefs and values that promote tradition, certainty, consensus and clarity (Jost *et al.*, 2003). Indeed, a variety of discrete threats, including economic threat, shift people toward more fervent belief in nationalism, ingroup superiority and authoritarianism (Hogg, 2000; McGregor *et al.*, 2001; Nail *et al.*, 2009; Hogg *et al.*, 2013). Additionally, these types of beliefs are reliably associated with dorsal ACC and lateral PFC structure and function. For example, conservative ideology is associated with reduced N2 mean amplitude (Weissflog *et al.*, 2013), reduced ERN mean amplitude (Amodio *et al.*, 2007) and reduced ACC cortical volume (Kanai *et al.*, 2011). Similarly, religious belief is associated with reduced ERN mean amplitude (Inzlicht *et al.*, 2009), and group-focused moral beliefs are associated with reduced cortical volume in both the dorsal ACC and lateral PFC (Nash *et al.*, 2017). Following from this research, we conducted exploratory analyses to test whether increased dorsal ACC activation would mediate socio-economic attitude change elicited by real-world economic threat. To this end, we created a measure of wealth justification to index a relevant socio-economic belief. We reasoned that participants may seek to quell angst about money by exaggerating support and motivation for personal wealth. Alternatively, economic threat may inspire motivated social cognition for the certainty and clarity provided by normative capitalistic beliefs (Jost *et al.*, 2003). Critically, if support for personal wealth represents a kind of conflict resolution, then it should be driven by heightened ACC activation to conflict. This finding could potentially give insight into the emotional and social consequences of real-world economic threat.

## Method

### Participants

Ethical approval for this study was provided by the University of Alberta Human Research Ethics Board (Protocol 00084513). Participants (*N* = 110; modal age = 19; age range = 17–26; females = 61) with normal or corrected-to-normal vision were recruited from a first-year psychology class and earned class credit. Based on pilot data indicating that the current manipulation had a medium to large effect size on anxious uncertainty (Cohen’s *d* = 0.65), we aimed to include 50 individuals per condition and stopped collection at the end of the 2019 fall term (power analyses in G*Power: difference between two independent groups, ‘expected’ effect size *d *= 0.65, alpha = 0.05, power = 0.80, and number of groups = 2, total sample size = 60). A total of 7 participants were excluded due to poor connectivity (as indicated by impedances > 10 kOhms, *N* = 5) or missing EEG data (*N* = 2), leaving 103 participants for analyses.

### Procedure

Participants first completed an electronic informed consent, then were fitted with a 64-channel EEG headset (Brain Products) and seated at a computer station in an electrically and sound-shielded room. All materials were completed on a computer and in the following order. Participants first answered demographic questions and several personality questionnaires as part of a larger research project on individual differences in the neuroscience of self-regulation (all data available upon request). Participants were then randomly assigned to either the ‘Economic Threat’ condition or the ‘No-Threat Control’ condition. Participants then completed the passive auditory oddball task. Again, as part of a separate line of research, participants then completed a color-naming Stroop task, and a Balloon Analog Risk-Taking task (manuscripts in prep.). After, participants completed the wealth justification scale. Finally, participants rated the degree to which the economic threat manipulation made them feel a range of different positive and negative emotions, including, in order, Good, Happy, Smart, Successful, Likeable, Meaningful, Frustrated, Confused, Uncertain, Empty, Anxious, Ashamed, Insecure, Lonely, Stupid, Out of Control and Angry (1 = Strongly Disagree, 5 = Strongly Agree). They then completed a measure of conscientiousness. Participants were then debriefed, had the headset removed and hair washed, and thanked for their time.

### Economic threat manipulation

Participants in the Economic Threat condition read an ostensibly real article from CBC.ca about an unsettling economic forecast in Canada that would specifically impact young adults. The forecast was putatively compiled by top Canadian researchers who concluded that a recession was imminent and that students would be hit hardest given the vulnerable position they were left in by the 2008 economic crisis. As such, this article was tailored to participants in our sample, i.e. young students. Participants in the No-Threat Control condition read an ostensibly real article from CBC.ca about a more neutral economic forecast that emphasized stability and a continuation of the status quo. Notably, both forecasts were based on real, publicly available economic predictions from financial news outlets.

### Passive auditory oddball paradigm

After the manipulation, participants listened to a series of standard tones (pure 1000 Hz tones for 50 ms) and white noise blasts (0–20 000 Hz ‘hissing’ sound for 50 ms, both at volume setting 50 in Windows) presented at 75 and 80 dB SPL, respectively. The ratio of white noise to standard tones was 1:9. A stimulus was presented each second and the entire paradigm lasted for three and a half minutes, for a total of 210 trials (approximately 21 white noise and 189 standard tone trials). Participants were informed that they would hear noises but were not instructed to do anything other than fixate on a small circle presented on the computer screen during the task. Importantly, the passive auditory oddball task allows more basic conflict detection processes to be probed without interference from task-based processing in the dorsal ACC.

### Wealth justification scale

We constructed a 12-item wealth justification scale to measure the degree to which people support and justify personal wealth. This scale included items such as ‘The amount of money I make is important to me,’ ‘Accumulating wealth is one of my main goals in life’ and ‘Getting rich usually means stepping on others to get there. (reverse coded).’ Reliability analysis revealed that two items did not correlate with the scale item total and were removed. This 10-item scale demonstrated acceptable reliability, Cronbach’s alpha = 0.745. We thus created a composite wealth justification score from the final 10 items. Note that mediation effects are the same with the full 12-item scale.

### EEG recording and preprocessing

**Fig. 1.**
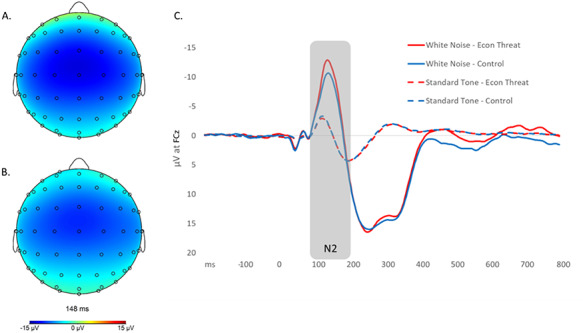
Topography of the N2 peak after white noise stimulus presentation at FCz in (A) Economic Threat and (B) No-Threat Control conditions (peak latencies Economic Threat = 145 ms; No-Threat Control = 148 ms). (C) Grand average ERP at FCz to white noise and standard tone stimuli in both the Economic Threat and No-Threat Control conditions.

Continuous EEG was recorded using the 64 Ag-AgCl channel ActiCHamp EEG system (Brain Products), positioned according to the 10/10 system and digitized at a sampling rate of 512 Hz (24 bit precision; bandwidth: 0.1–100 Hz). During recording, signals were referenced to TP9 electrode positioned over the left mastoid. Offline, EEG was re-referenced to the average mastoids (TP9-TP10), down-sampled to 256 Hz, band-pass filtered between 0.1 and 30 Hz, and notch filtered at 60 Hz. Blinks were statistically removed using the automatic ocular correction developed by Gratton and Coles (1993). Artifacts were then automatically detected using the following parameters: −100 to +100 μV min/max threshold, 50 μV maximum voltage step, 0.5 μV lowest allowed voltage (maximum–minimum) in 100-ms intervals. Data were segmented into 1000-ms epochs locked on either standard tone or white noise presentation, 200 ms before to 800 ms after the stimulus. For each participant, all artifact-free epochs were then baseline-corrected by subtracting the average voltage during the −200–0 ms time period prior to the stimulus and averaged, creating average ERPs of standard tone (average per participant = 184.29) and white noise (average per participant = 20.58). The N2 was defined for both standard tones and white noise stimuli as the mean negative amplitude between 100 and 200 ms after stimulus at the site where the startle component was maximal, the fronto-central electrode FCz (see grand average ERPs in Figure 1).

### Source localization of conflict detection processes

We used sLORETA to estimate the cortical sources of scalp-recorded activity during conflict detection across economic threat conditions. As opposed to dipole modelling, sLORETA computes activity as current density (A/m^2^) without assuming a predefined number of active sources. The sLORETA solution space consists of 6239 voxels (voxel size: 5 × 5 × 5 mm) restricted to cortex and hippocampi, as defined by the digitized Montreal Neurological Institute (MNI) probability atlas. sLORETA has been reliably validated by research comparing sLORETA localization of EEG activity to fMRI data (Mobascher *et al.*, 2009; Olbrich *et al.*, 2009), Positron Emission Tomography data (Laxton *et al.*, 2010) and implanted electrodes in intracranial recordings (Zumsteg *et al.*, 2006).

For each participant, sLORETA images were computed for scalp-recorded activity for both the white noise and standard tone average ERPs. These images were normalized to a total current density of one and log-transformed.

### Statistical analyses

Preliminary analyses examined if N2 mean amplitudes (100 ms–200 ms) were impacted by the economic threat manipulation. We conducted an independent-samples *t*-test with the condition variable entered as the grouping variable and a difference score between white noise N2 mean amplitude at FCz minus standard tone N2 mean amplitude at FCz as the dependent variable. One participant is excluded from these mean amplitude analyses for not demonstrating an N2 at FCz (inclusion or exclusion of this participant does not change our primary results).

For our main analyses, to isolate conflict detection processes during the N2 component, all images were baseline corrected for pre-stimulus activation (−200-0 ms). In order to more precisely examine conflict detection processes, our analyses focused on the paired contrast between white noise and standard tone sLORETA images. Thus, the standard tone sLORETA images were subtracted from the white noise sLORETA images during analyses to remove processes common to both stimuli, allowing more isolated focus on conflict detection to white noise. Whole-brain voxel-by-voxel independent groups *t*-tests of the sLORETA images were conducted on the timeframe during the N2 component, or 100–200 ms after stimulus presentation as seen in the grand average N2 (Figure 1), thus comparing intracerebral sources of conflict detection in the Economic Threat and the No-Threat Control conditions. Correction for multiple testing for all 6239 voxels was implemented by means of a nonparametric randomization approach (Nichols and Holmes, 2002). This approach estimates empirical probability distributions and the corresponding critical probability thresholds (corrected for multiple comparisons). We expected that the economic threat manipulation would cause increased activation in the dorsal ACC during the N2 timeframe, indicating heightened sensitivity to conflict. However, our whole brain approach allowed for examining other potential differences in intracerebral sources across conditions.

Given that the dorsal ACC is thought to recruit the lateral PFC to regulate conflict or implement cognitive control (Botvinick *et al.*, 2004; Mansouri *et al.*, 2009; Miller and Cohen; 2001; Yeung *et al.*, 2004), we also conducted exploratory analyses focused on subsequent components in the ERP to white noise. To determine if heightened dorsal ACC activation during the N2 timeframe caused by economic threat leads to increased lateral PFC activation, we used a more focused small-volume analysis corrected for only voxels in lateral PFC regions (see Gianotti *et al.*, 2018 for such an approach). We explored both the P3 and the late positive potential (LPP) timeframes—i.e. two components important in emotional arousal and emotion regulation (Hajcak *et al.*, 2010). The P3 timeframe was determined as 200–400 ms after stimulus at the site where the component demonstrated the maximal mean amplitude, Cz. The LPP timeframe was determined as 480–620 ms after stimulus, maximal at Pz.

## Results

As can be seen in (Figure 1A and B,) the N2 topography was largely similar across Economic Threat and No-Threat Control conditions, with maximal amplitude at the FCz electrode, and similar peak latencies after white noise stimulus presentation (Economic Threat = 145 ms; No-Threat Control = 148 ms). An independent-samples *t*-test revealed that participants in the Economic Threat condition, compared to the No-Threat Control condition, demonstrated a non-significant trend towards larger (i.e. more negative) N2 mean amplitude difference scores (white noise—standard tone) at FCz, *t*(100), = 1.621, *P* = 0.108 (equal variances not assumed, *t*(99.998), = 1.666, *P* = 0.099). However, our primary hypotheses were focused on source-localized activation during the N2 to more precisely target conflict detection processes.

**Table 1.** Coordinates for active voxels for whole brain and small volume corrections

Timeframe	Structure	Brodmann area	MNI coordinates (*x, y, z*)	*t*-value
N2^a^	Anterior cingulate	32	−5, 45, 15	4.861
		32	−5, 40, 20	4.640
		32	−5, 40, 15	4.470
		32	−5, 35, 20	4.349
	Medial frontal gyrus	10	−5, 50, 15	4.730
		9	−5, 45, 20	4.552
		10	0, 55, 10	4.518
		10	−5, 50, 10	4.351
		9	−10, 40, 20	4.278
		10	−5, 55, 15	4.230
		10	−5, 60, 10	4.227
LPP^b^	Inferior frontal gyrus	9	−45, 5, 35	3.200
	Middle frontal gyrus	9	−40, 12, 36	3.240
	Precentral gyrus	9	−35, 12, −36	3.120

a Whole-brain corrected threshold *t* = 4.175.

b Small-volume (lateral PFC) corrected threshold *t* = 3.114.

**Fig. 2.**
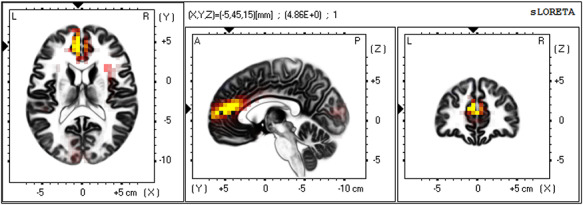
Source localization results showing voxels with significantly higher activation in the Economic Threat condition than in the No-Threat Control condition in the dorsal ACC during the N2 component, whole-brain corrected. Significant voxels in yellow, critical *t*-value > 4.175. Arrows at peak voxel, MNI coordinates = −5, 45, 15, *t*(101) = 4.860.

**Fig. 3.**
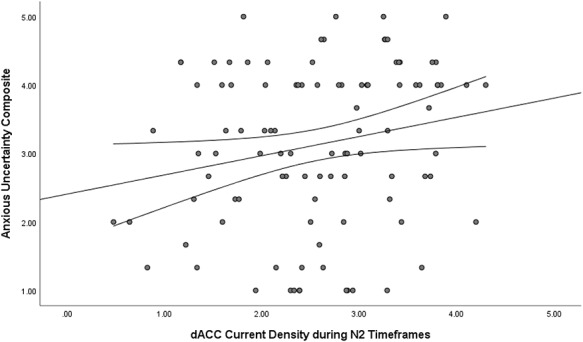
Scatterplot of the relation between dorsal ACC activation during the N2 and the anxious uncertainty composite.

Source localization analyses revealed that a cluster of 11 voxels in the dorsal ACC/medial PFC demonstrated significantly higher activation in the Economic Threat condition than in the No-Threat Control condition (see Table 1 and Figure 2), *t*-value threshold corrected for multiple comparisons = 4.175. The peak voxel was located in Brodmann Area 32 of the dorsal ACC, MNI coordinates = −5, 45, 15, *t*(101) = 4.860. The cluster also included voxels spanning into Brodmann Areas 9 and 10. No other voxels exceeded the *t*-value threshold corrected for multiple comparisons. To further characterize this pattern of activation, we extracted individual estimates of current density across voxels in a 10-mm sphere around the peak voxel in the dorsal ACC during the same N2 timeframe (i.e. 100–200 ms after stimulus presentation) and correlated (Pearson correlation, two-tailed) these scores with an anxious uncertain composite of self-reported affect to the economic manipulations. First, a one-way ANOVA revealed that the Economic Threat condition caused increased anxious uncertainty (*M* = 3.719, *SD* = 0.968), compared to the No-Threat Control condition (*M* = 2.389, *SD* = 1.027), *F*(1, 101) = 44.981, *P* < 0.0001. We found that dorsal ACC activation during the N2 was positively correlated with the anxious uncertainty composite, *r* = 0.226, *P* = 0.022 (Figure 3). This further supports the idea that economic threat, as an anxiety-provoking and uncertain experience, caused increased conflict detection sensitivity (see S1 in the Supplementary Materials for further analyses using positive and negative affect composites).

We next conducted an exploratory mediation analysis to examine if dorsal ACC activation during the N2 mediated an effect of economic threat on the wealth justification scale. Bootstrapped mediational analyses (PROCESS model 4, 5000 bootstrap samples for 95% confidence intervals, see Hayes, 2017) were conducted in which the predictor (X) was the Economic Threat manipulation variable, the mediator (M) was dorsal ACC activation during the N2 and the dependent variable (Y) was the composite wealth justification score. Results showed that the Economic Threat condition caused increased dorsal ACC activation during the N2 and this dorsal ACC activation was associated with increased wealth justification, indirect effect coefficient = 0.4367, *SE* = 0.2788, 95% CI [0.0012, 1.0949] (CIs do not contain zero). These results demonstrate that economic threat increases dorsal ACC activation during conflict detection, which in turn boosts wealth justification (see S2 in the Supplementary Materials file).

**Fig. 4.**
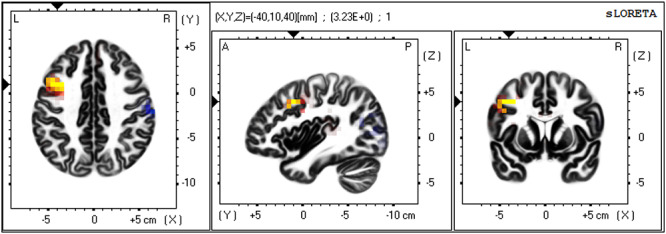
Source localization results showing voxels with significantly higher activation in the Economic Threat condition than in the No-Threat Control condition in the left dlPFC during the LPP component, small-volume corrected. Significant voxels in yellow, critical *t*-value > 3.114. Arrows at peak voxel, MNI coordinates = −40, 10, 40, *t*(101) = 3.230.

Finally, exploratory analyses were conducted to focus on the lateral PFC. We restricted the voxel-by-voxel independent-samples *t*-tests of sLORETA images to Brodmann Areas classically associated with lateral PFC regions (Brodmann Areas 9, 10, 45, 46 and 47) during the following timeframes, 200–400 ms (the P3) and 480–620 ms (the LPP). Correction for multiple testing was thus calculated for 862 voxels using the same nonparametric randomization approach (Nichols and Holmes, 2002). Results showed that a cluster of 3 contiguous voxels in the left dorsolateral PFC (dlPFC) demonstrated significantly higher activation in the Economic Threat condition than in the No-Threat Control condition during the LPP timeframe, *t*-value threshold corrected for multiple comparisons = 3.114 (see Table 1 and Figure 4). The peak voxel was located in Brodmann Area 9, MNI coordinates = −40, 10, 40, *t*(101) = 3.240. As in the N2 analyses, we again extracted individual estimates of current density across voxels in a 5 mm sphere around the peak voxel in the left dlPFC during the LPP timeframe. We conducted mediation analysis (PROCESS model 4, 5000 bootstrap samples for 95% confidence intervals, covariate: baseline left dlPFC activation, see Hayes, 2017) to examine if dorsal ACC activation during the N2 mediated the effect of economic threat on left dlPFC activation. Contrary to expectations, however, mediation analyses revealed an opposing indirect effect, such that increased dorsal ACC activation during the N2 timeframe due to the economic threat manipulation mediated a decrease in left dlPFC activation during the LPP timeframe, (indirect effect coefficient = −0.1753, *SE* = 0.1072, 95% CIs [−0.4217, −0.0058]). This suggests that increased conflict detection sensitivity caused by Economic Threat appears to disrupt downstream processes instantiated in the lateral PFC. Note that no other voxels during the late positive potential and no voxels during the P3 timeframe exceeded this small-volume *t*-value threshold corrected for multiple comparisons.

## Discussion

Economic threat is a poignant experience with profound emotional and social effects (e.g. Roszkowski and Davey, 2010; Karanikolos *et al.*, 2013; McInerney *et al.*, 2013; Buffel *et al.*, 2015; Kriesi and Pappas, 2015; Aslanidis, 2016; Bianchi, 2016; Margerison-Zilko *et al.*, 2016; Ybarra *et al.*, 2016). Widespread economic threat looms again today in the context of the COVID-19 pandemic. A better understanding of the underlying neurocognitive mechanisms may help shed light on the broader social and emotional effects of economic threat. To that end, we focused on conflict detection processes. We found that economic threat caused increased activation source localized to the ACC during the N2 component to white noise stimuli. Increased activation in the ACC was also correlated with increased self-reported anxious uncertainty. Further, economic threat, mediated by ACC activation during the N2, increased personal wealth justification.

The current findings extend prior research on threat and neurocognitive mechanisms in a key way. The economic threat was a poignant, generalizable experience with important psychological consequences, whereas prior research has typically manipulated discrete and temporary threats during the task that elicit conflict and the N2 in a within-subject design. Manipulating anxiety or threat and conflict concurrently in the same task can sometimes reduce conflict detection sensitivity, reflecting divided attentional resources (Pessoa, 2009). To avoid processes such as divided attention and directly focus on the causal, downstream effects of a real, pervasive and anxiety-provoking threat, we separated the threatening experience from the measure of conflict.

Our findings also compliment previous research that has commonly linked conflict detection and resolution to a dorsal ACC-lateral PFC network in which the dorsal ACC identifies conflict or uncertainty in a current goal or task and recruits the lateral PFC to resolve the conflict or implement cognitive control (Botvinick *et al.*, 2004; Mansouri *et al.*, 2009; Miller and Cohen; 2001; Yeung *et al.*, 2004). Importantly, negative affect is processed in the dorsal ACC as a signal of conflict or the need for increased cognitive control (Shackman *et al.*, 2011). Alternatively, anxiety is associated with limited control capacity (Eysenck *et al.*, 2007). This raised the possibility that increased ACC activation to conflict after economic threat had downstream consequences for lateral PFC function. A more focused correction procedure (e.g. corrected for only voxels in lateral PFC regions) revealed increased left dlPFC activation during a subsequent component in the ERP, the LPP. Further, contrary to expectations, dorsal ACC activation during the N2 timeframe caused by the economic threat manipulation mediated a decrease in left dlPFC activation during the LPP timeframe. Because the LPP component is related to emotional arousal and emotion regulation (Hajcak *et al.*, 2010), and the dlPFC is related to emotion regulation and cognitive control (among other processes), we may speculate that the increased conflict detection sensitivity disrupts downstream emotion regulation processes (e.g. Bishop *et al.*, 2004). Consistent with this, prior research has demonstrated that increased working memory load, a process that activates the dlPFC, reduces LPP amplitude unless participants reported higher levels of anxiety (MacNamara *et al.*, 2011). That is, anxiety disrupted emotion regulation processes that involve the dlPFC.

Relatedly, increased sensitivity to conflict instantiated in the ACC may be an underlying neural mechanism in the link between economic threat and increased stress, anxiety and depression on the one hand, and increased authoritarianism, prejudice and nationalism on the other. For example, a pervasive sense of economic threat may lead to chronically higher sensitivity to conflict, emotional dysregulation and chronically higher levels of anxious arousal. Chronic anxiety and distress can lead to depression (Gray and McNaughton, 2000) and negative consequences for an array of neurobiological processes (Rodrigues *et al.*, 2009; Roozendaal *et al.*, 2009; Ulrich-Lai and Herman, 2009).

Additionally, we found that ACC activation during conflict mediated an increase in positive attitudes toward personal wealth accrual, possibly because participants were motivated to bolster support of the capitalistic system (Jost *et al.*, 2003; Kay *et al.*, 2009) in order to resolve conflict aroused by the economic threat. Alternatively, this could also reflect a simpler kind of conflict resolution, in which angst about money is resolved by exaggerated support for personal wealth. We anticipate that this exploratory finding will encourage future research examining the causal link between real-world economic threat and conflict resolution processes. For example, our study was limited in that it did not assess the extent to which support for personal wealth served to quell negative affect caused by economic threat. Future work could probe the emotion regulation effects of these and other proposed conflict resolution processes. Researchers could further examine if increased sensitivity to conflict and dorsal ACC activation mediates the broader and more varied changes in mental health outcomes and changes in ideology and belief associated with economic threat.

Despite the widespread impact of economic threat, its basic neurocognitive functions have remained unclear. Our results provide novel experimental evidence that economic threat activates conflict detection processes source localized to the ACC. Further, this study shows that these conflict detection processes can have downstream influence on attitude change relevant to economic threat, such as support for personal wealth. Finally, this study provides an important initial contribution toward future research examining the broader social and emotional effects of economic threat.

## Supplementary Material

nsaa139_Supp
