# Supplementary material for: Economic threat heightens conflict detection: sLORETA evidence
Source: Soc Cogn Affect Neurosci. 2020 Oct 7;15(9):981–90. doi: 10.1093/scan/nsaa139 (PMC7647378; doi:10.1093/scan/nsaa139)
Supplement: nsaa139_Supp [file nsaa139_supp.zip › nsaa139_scan-20-193-File007.docx]

**Supplementary Material** **- *Economic Threat Heightens Conflict Detection: sLORETA evidence***

**S1.** We computed two more composites, a positive affect composite (items: Good, Happy, Smart, Successful, Likeable, and Meaningful) and a negative affect composite (items: Frustrated, Confused, Empty, Ashamed, Insecure, Lonely, Stupid, Out of Control, and Angry). Next, a one-way ANOVA revealed that the Economic Threat condition caused increased negative affect (*M* = 3.719, *SD* = .968), compared to the No-Threat Control condition (*M* = 2.389, *SD* = 1.027), *F*(1, 101) = 29.368, *p* < .0001. Similarly, a second one-way ANOVA revealed that the Economic Threat condition caused decreased positive affect (*M* = 2.290, *SD* = .547), compared to the No-Threat Control condition (*M* = 3.308, *SD* = .684), *F*(1, 101) = 70.407, *p* < .0001. However, if anxious uncertainty is entered as a covariate, the effect of condition on negative effect is no longer significant, *F*(1, 101) = 1.008, *p* = .318, whereas the effect on positive affect remains significant, *F*(1, 101) = 28.103, *p* < .0001. Finally, if negative affect or positive affect are entered as covariates, the condition effect on anxious uncertainty remains significant, *F*(1, 101) = 70.407, *p* < .0001. This suggests that the manipulation specifically increased anxious uncertainty and not a more general negative affect.

Finally, we correlated these two additional composites with dorsal ACC activation during the N2. We found that neither the negative affect composite (*r* = .095, *p* = .339) nor the positive affect composite (*r* = -.084, *p* = .397) were significantly associated with dorsal ACC activation. Thus, only the anxious uncertainty composite was correlated with dorsal ACC activation during the N2 timeframe.

**S2.** Mediation analyses were deemed inappropriate for the anxious uncertain composite given that it was completed by participants at the end of the study but indexes anxious uncertainty retrospectively, i.e., prior to the auditory oddball task. On the other hand, the wealth justification mediation analysis was not complicated by incongruency between temporal and conceptual order. As such, we did not test mediation for the anxious uncertainty composite, nor correlation for the wealth justification.
